# Effect of Goreisan, a Japanese Traditional Medicine, on Cortical Spreading Depolarization in Mice

**DOI:** 10.3390/ijms232213803

**Published:** 2022-11-09

**Authors:** Chisato Iba, Miyuki Unekawa, Yoshikane Izawa, Jin Nakahara, Tsubasa Takizawa

**Affiliations:** Department of Neurology, Keio University School of Medicine, Tokyo 160-8582, Japan

**Keywords:** Goreisan, Kampo medicine, cortical spreading depolarization, migraine

## Abstract

Goreisan, a traditional Japanese Kampo medicine, is often used to treat headaches, including migraines; however, the underlying mechanisms remain unknown. Therefore, we investigated whether chronic treatment with Goreisan affects cortical spreading depolarization (CSD) in migraines. CSD susceptibility was assessed in male and female C57BL/6 mice by comparing CSD threshold, propagation velocity, and CSD frequency between animals treated with Goreisan for approximately 3 weeks and the corresponding controls with a potassium-induced CSD model. No significant differences were observed in CSD susceptibility between mice that were chronically treated with Goreisan and the control mice. Additionally, no significant differences were observed in other physiological parameters, including body weight, blood gases, and blood pressure. CSD susceptibility was not affected by chronic treatment with Goreisan, which suggests that the drug treats headaches via mechanisms that do not involve CSD modulation.

## 1. Introduction

Migraine is one of the most prevalent neurological disorders worldwide, affecting over a billion people [1], with a global prevalence of approximately 10% (6% for males and 14% for females) and a varied geographical distribution. The disease occurs more frequently in Europe (15%) and North America (13%) than in Asia (9%) and Africa (5%) [2,3].

Acetaminophen, nonsteroidal anti-inflammatory drugs, triptans, ditans, and gepants are routinely recommended for the treatment of acute migraines. Furthermore, prophylactic drugs such as valproate, amitriptyline, gepants, and anti-CGRP monoclonal antibodies are used. Additionally, Japanese traditional medicines, called Kampo medicines, are often used to treat headaches in Japan. One such Kampo medicine, Goreisan, has been traditionally used to treat headaches, including migraines. Previous studies have reported the utility of Goreisan, a mixture of Alismatis Tuber, Atractylodis Lanceae Rhizoma, Polyporus, Poria, and Cinnamomi Cortex [4], in the treatment of migraines related to weather changes [5]. The herbs mentioned above act antagonistically on vasopressin receptors and exert diuretic and anti-inflammatory effects while relieving dysuria and edema and promoting blood capillary circulation. Although Goreisan is documented in the “Clinical Practice Guidelines for Headache 2021”, published by the Japanese Headaches Society [6], the mechanisms through which it alleviates headaches are poorly understood. 

Cortical spreading depolarization (CSD) is a slowly propagating depolarization wave with well-recognized roles in migraine pathology. Consequently, experimental models of CSD have been established as migraine models [7]. CSD is a biological event of the aura phase of migraines, which is known to generally occur for 5–60 min before headache; 25% of migraineurs have an aura, and the most frequent type is a visual aura. In 2001, Hadjikhani et al. [8] captured a CSD-like wave in migraineurs using functional magnetic resonance imaging (fMRI). Patients with migraine with aura were subjected to fMRI during the aura phase. The change in the blood oxygenation level-dependent (BOLD) signal in the occipital cortex contralateral to the visual aura resembled the CSD observed in an animal model [7,8]. Regarding CSD, there is a slight difference between rodents and humans. In rodents, as there is no sulcus, CSD spreads through the entire ipsilateral cortex. In contrast, in humans, as CSD does not spread beyond the sulcus, it is considered that CSD is localized to the occipital lobe in patients with migraine with a visual aura. Although the CSD model is an established in vivo migraine model, it remains unknown whether CSD occurs in patients with migraine without aura. In migraines with a “silent aura”, CSD may occur in the cortex without neurological functions. Here, we examined the effects of Goreisan on CSD in a mouse migraine model to investigate its mechanism of action in patients with migraine. CSD susceptibility has been used to test the pharmacological effects of different agents against migraine [7,9]; furthermore, Liu et al. showed that a novel treatment in the field non-invasive vagus nerve stimulation has the potential to inhibit CSD [10]. We selected the CSD model as a reliable migraine model to confirm the effect of Goreisan on migraine. 

Goreisan has been reported to decrease aquaporin 4 (AQP4) mRNA expression in a hypoxic–ischemic encephalopathy rat model [11] and the AQP4 protein level in a middle cerebral artery occlusion mouse model. AQP4 is expressed in perivascular and submucosal astrocytes and is involved in the homeostatic transport of water from blood vessels to the brain parenchyma and spinal fluid space. A previous study reported that the velocity and frequency of CSD are low in AQP4-deficient mice [12]. Furthermore, in clinical practice, Goreisan is often used in “Suidoku”, which means “conditions with water imbalance” in Japanese, such as edema and dehydration, resembling the involvement of AQP4. We speculated that Goreisan targets CSD via AQP4.

Some studies have reported that long-term treatment with medicines such as topiramate and valproate inhibits CSD sensitivity in rodents [7,9]; therefore, we administered Goreisan to mice for approximately 3 weeks. Additionally, the effects of Goreisan on body weight, blood gases, and electrolytes were monitored.

## 2. Results

**Figure 1:**
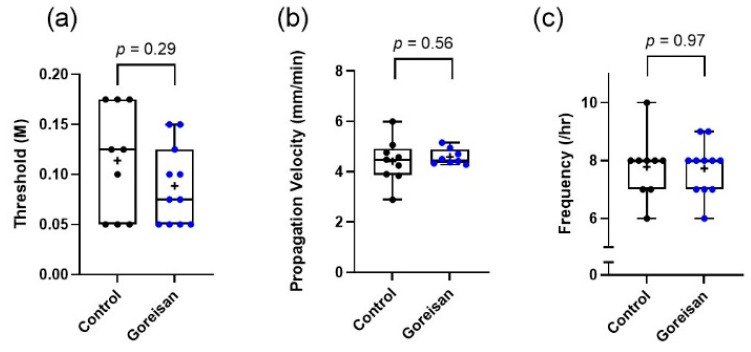
Comparison of CSD susceptibility between the Goreisan and control groups. No significant differences in CSD (**a**) threshold, (**b**) propagation velocity, and (**c**) frequency were observed between the Goreisan and control groups. Statistical analyses were performed using the Wilcoxon signed-rank test.

A total of 11 mice in the Goreisan group (seven male and four female mice) and 9 mice in the control group (six male and three female mice) were included in the study. There was no difference in the number of animals excluded due to physiological problems and procedural difficulties between the groups (seven from the Goreisan group and nine from the control group). The median (range) of the CSD threshold (M) was 0.075 (0.050–0.125) in the Goreisan group and 0.125 (0.050–0.175) in the control group (*p* = 0.29). The propagation velocity (mm/min) was 4.5 (4.3–4.9) and 4.5 (3.9–4.9) (*p* = 0.56), and the frequency (per 60 min) was eight (seven to eight) and eight (seven to eight) (*p* = 0.97), respectively, in the Goreisan and control groups. No significant differences were observed in the CSD threshold, propagation velocity, frequency, and other CSD indicators between the Goreisan and control groups. A comparison of the CSD susceptibility between the groups is depicted in Figure 1. 

Although the [Na^+^] observed in the blood gas test tended to be lower in the Goreisan group before CSD induction than in the control group, the difference was not significant. Other indicators assessed in the blood gas test, blood pressure and heart rate, both pre- and post-CSD induction, were also not significantly different between the groups (Table 1). Additionally, the changes in body weight over the 3-week period were not significantly different. Furthermore, no significant differences were observed between the male and female mice with respect to the mentioned CSD parameters. CSD was not induced by 1 M KCl in mice (n = 2) who had been intraperitoneally injected with MK801 (positive control for CSD inhibition).

## 3. Discussion

Our results demonstrated no significant differences in CSD susceptibility between the Goreisan-treated and control mice over a 3-week treatment regimen. This implies that Goreisan intake does not attenuate CSD susceptibility in a potassium-induced CSD mouse model. Therefore, mechanisms of action that do not involve CSD modulation are likely to play a role in the Goreisan-mediated alleviation of migraines. As CSD is considered to be involved in the pathology of migraines with auras, it is possible that Goreisan has an effect on patients with migraines without auras. Whether Goreisan has an effect on migraines with or without auras has not been studied; thus, further clinical studies are necessary.

A previous study reported that the velocity and frequency of CSD are low in aquaporin 4 (AQP4)-deficient mice [12]. Additionally, other evidence suggests that Goreisan treatment improves ischemic brain edema and stroke in rodents [11,13], which indicates that the medication may cross the blood–brain barrier to reach the brain. Furthermore, Goreisan reduces AQP4 expression [11]. Consequently, we tested the hypothesis that Goreisan may exert inhibitory effects on CSD, which was, however, disproven by our results.

In the present study, the KCl method was used to induce CSD. KCl may damage the cortical tissue; a less invasive method to induce CSD may be optogenetics [14]. Although the conventional KCl method has been established and is widely used, in order to perform CSD experiments less invasively, transgenic mice expressing channelrhodopsin-2 in neurons (Thy1-ChR2-YFP) and an optogenetic setup would be more ideal.

Recently, there have been reports on several molecular targets in the pathophysiology of migraine such as calcitonin gene-related peptide (CGRP), pituitary adenylate cyclase-activating peptide (PACAP), nitric oxide (NO), transient receptor potential cation channel subfamily vanilloid 1 (TRPV1), and transient receptor potential cation channel subfamily melastatin 8 (TRPM8) [15,16]. To our knowledge, no study has focused on Goreisan and the above molecules. Although Goreisan did not affect CSD susceptibility, to identify the potential mechanism by which Goreisan alleviates headache, future research should focus on the above molecules as potential targets. Furthermore, CSD is known to cause neuroinflammation and neuronal activation in ipsilateral cortex, such as the upregulation of interleukin 1 beta (IL-1β), tumour necrosis factor alpha (TNFα), and c-Fos [17,18]. Whether Goreisan has an inhibitory effect downstream of CSD, for example, whether it suppresses the increase in cytokine or c-Fos expression, remains to be further studied.

This study has several limitations. Generally, the level of drug effect in humans is converted using body surface area based on the level of drug effect in experimental animals. The dose of Goreisan used in our study was approximately four times more than the dose used in humans. Furthermore, a previous study on Goreisan used 0.5% or 1.0% doses [19]; thus, we selected 1.0% in our experiment. Although a 3-week treatment at the above dosage seemed to be reasonable considering previous CSD experiments with prophylactic drugs [9], an increase in the dose and/or duration of Goreisan administration may affect certain CSD parameters. Furthermore, investigating CSD in other species, such as rats, may be informative, as several previous studies on the relationship between migraine drugs and CSD have been conducted in rats rather than in mice [7].

## 4. Materials and Methods

### 4.1. Experimental Procedures

All experimental procedures were approved by the Keio University Institutional Animal Care and Use Committee (authorization no. 20006 and A2021-006) and were performed in accordance with the university’s guidelines and the ARRIVE (Animal Research: Reporting In Vivo Experiments) reporting guidelines for the care and use of laboratory animals.

C57BL/6 mice were purchased from CLEA Japan Inc. (Tokyo, Japan). The mice were housed under a 12 h dark–light cycle with free access to water and food, and body weight was measured twice a week at around 9 A.M. 

**Figure 2:**
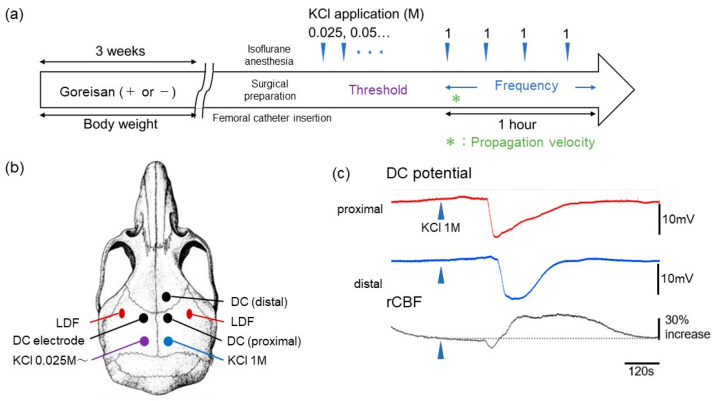
Experimental protocol and cortical spreading depolarization (CSD) setup. (**a**,**b**) C57BL/6 mice (males: 5 weeks old, females: 7 weeks old) were fed either normal chow (100% MF, control group) or chow supplemented with 1% Goreisan (1% Goreisan + 99% MF, Goreisan group) for approximately 3 weeks. (**c**) Representative image of the DC potential and regional cerebral blood flow (rCBF) changes during CSD. DC: Direct current, LDF: laser Doppler flowmeter.

The experimental protocol and setup for measuring CSD susceptibility are depicted in Figure 2. We used 24 male mice (5 weeks old) and 12 female mice (7 weeks old), and the animals were randomly divided into two groups. The mice in the control group were administered normal chow (MF 100%; Oriental Yeast Co., Ltd, Tokyo, Japan), whereas those in the experimental group were fed chow supplemented with Goreisan (MF 99% + Goreisan 1%) (Tsumura & Co., Tokyo, Japan) for approximately 3 weeks. The dose (1%) of Goreisan mixed in normal chow was determined based on previous studies [19,20]. The investigator was blinded to the type of treatment administered to the mice.

On the day of the experiment, the mice were anesthetized using isoflurane (2.0%) and cannulated in the femoral artery. All procedures were performed with simultaneous blood pressure monitoring (surgical strain gauge and BP amplifier; MLT0670 and ML117, ADInstruments, Ltd., Sydney, Australia) and maintenance of rectal temperature at 37 °C using a heating pad (BWT-100A; Bioresearch Center Co., Nagoya, Japan). The mice were fixed to a head-holder (SGM-4; Narishige Scientific Instrument Laboratory, Tokyo, Japan), as shown in the experimental setup in Figure 2b. Laser Doppler flowmeter (LDF; ALF 21, Advance Co., Ltd., Tokyo, Japan) probes (BF52; Advance Co., Ltd.) were affixed to the skull with dental cement to monitor regional cerebral blood flow (rCBF). Five holes of approximately 1 mm diameter were subsequently drilled: posterior holes (4 mm posterior and 2 mm lateral to the bregma) for KCl induction and parietal (±2 mm lateral and 2 mm caudal to the bregma) and frontal holes (2 mm lateral and 2 mm rostral to the bregma) for direct current (DC) potential recording. DC potentials were measured using a differential headstage and extracellular amplifier (Model 4002 and EX1; Dagan Co., Minneapolis, MN, USA). Three Ag/AgCl DC electrodes (tip diameter; 200 µm, EEG-5002Ag; Bioresearch Center Co., Ltd.) were placed on the dura at the proximal and distal holes prior to fixation with dental cement. DC potentials, rCBF, and blood pressure were recorded using a multichannel recorder (PowerLab 8/30; ADInstruments, Ltd., Sydney, Australia). DC potential and rCBF were recorded with LabChart 8 (ADInstruments, Ltd.). Arterial blood was drawn through the catheter for blood gas analysis (blood gas analyzer, RapidLab 348EX; Siemens AG, Munich, Germany) before and after CSD measurement

After confirming the nonoccurrence of CSD for at least 30 min, 5 µL of 0.025 M KCl solution was introduced into the left posterior hole. The KCl concentration was increased by 0.025 M at intervals of at least 5 min, and the concentration at which CSD occurred for the first time was considered the CSD “threshold” [21].

At least 10 min after CSD occurrence in the left hemisphere, a cotton ball (approximately 1 mm in diameter) soaked with 1 M KCl was placed in the right posterior hole, which was replaced every 15 min with a fresh ball. The number of CSD events induced in a 60 min period was counted as the “frequency.” The “propagation velocity” was calculated from the time differences and distances between the DC electrodes for the first CSD occurring in the right hemisphere.

To examine whether our experimental system accurately evaluated CSD susceptibility, MK801 (10 mg/kg BW; Sigma-Aldrich, St. Louis, MI, USA), an antagonist of the glutamate receptor N-methyl-D-aspartate and a well-known CSD inhibitor, was injected intraperitoneally 40 min before the measurement of CSD in separate C57BL/6 mice (11 weeks old, male, n = 2) as a positive control.

### 4.2. Statistics

All numeric data are expressed as mean (interquartile range). All analyses were performed using JMP 16 software (SAS Institute, Cary, NC, USA). Statistical analyses were carried out using the Wilcoxon-signed ranked test, and statistical significance was set at *p* < 0.05.

## 5. Conclusions

In conclusion, our study demonstrated no significant differences in CSD susceptibility between Goreisan-treated and control mice. The mechanisms by which Goreisan alleviates headaches are, therefore, unlikely to involve CSD modulation. Consequently, further studies are essential to elucidate the relationship between Goreisan administration and migraines.

## Figures and Tables

**Table 1:** Comparison of physiological parameters between the Goreisan and control groups. No significant differences in arterial blood gas, electrolytes, blood pressure, and heart rates were observed between the Goreisan and control groups before and after CSD measurement. MBP: mean blood pressure, Hct: hematocrit, HR: heart rate.

**Before CSD**	**pH**	**PaO_2_ (mmHg)**	**PaCO_2_ (mmHg)**	**HCO_3_ (mM)**	
Control	7.36 ± 0.05	106.3 ± 5.4	35.9 ± 3.2	19.8 ± 2.3	
Goreisan	7.36 ± 0.04	107.9 ± 8.0	36.2 ± 3.7	19.9 ± 1.9	
**Before CSD**	**Na^+^ (mEq/L)**	**K^+^ (mEq/L)**	**Hct (%)**	**MBP (mmHg)**	**HR (/min)**
Control	145 ± 4.4	4.00 ± 0.4	45 ± 2.8	86.2 ± 5.7	459 ± 44
Goreisan	141 ± 2.3	3.93 ± 0.4	47 ± 1.8	89.5 ± 8.9	473 ± 42
**After CSD**	**pH**	**PaO_2_ (mmHg)**	**PaCO_2_ (mmHg)**	**HCO_3_ (mM)**	
Control	7.33 ± 0.04	102.4 ± 11.7	43.7 ± 4.9	22.2 ± 3.0	
Goreisan	7.32 ± 0.04	105.9 ± 11.5	40.3 ± 3.2	20.5 ± 1.8	
**After CSD**	**Na^+^ (mEq/L)**	**K^+^ (mEq/L)**	**Hct (%)**	**MBP (mmHg)**	**HR (/min)**
Control	142 ± 2.3	4.68 ± 0.4	46 ± 2.4	76.3 ± 8.7	450 ± 76
Goreisan	140±3.3	4.67 ± 0.4	48 ± 4.0	76.8 ± 6.5	450 ± 29

## Data Availability

The data are available on reasonable request.
